# Bcl11b—A Critical Neurodevelopmental Transcription Factor—Roles in Health and Disease

**DOI:** 10.3389/fncel.2017.00089

**Published:** 2017-03-29

**Authors:** Matthew J. Lennon, Simon P. Jones, Michael D. Lovelace, Gilles J. Guillemin, Bruce J. Brew

**Affiliations:** ^1^Applied Neurosciences Program, Peter Duncan Neurosciences Research Unit, St. Vincent's Centre for Applied Medical ResearchSydney, NSW, Australia; ^2^Faculty of Medicine, St. Vincent's Clinical School, University of New South WalesSydney, NSW, Australia; ^3^Neuroinflammation Group, Faculty of Medicine and Health Sciences, Macquarie UniversitySydney, NSW, Australia; ^4^Departments of Neurology and Immunology, St. Vincent's HospitalSydney, NSW, Australia

**Keywords:** BCL11B, Ctip2, neurodevelopment, Huntingtons disease, Alzheimers disease, BDNF

## Abstract

B cell leukemia 11b (Bcl11b) is a zinc finger protein transcription factor with a multiplicity of functions. It works as both a genetic suppressor and activator, acting directly, attaching to promoter regions, as well as indirectly, attaching to promoter-bound transcription factors. Bcl11b is a fundamental transcription factor in fetal development, with important roles for the differentiation and development of various neuronal subtypes in the central nervous system (CNS). It has been used as a specific marker of layer V subcerebral projection neurons as well as striatal interneurons. Bcl11b also has critical developmental functions in the immune, integumentary and cardiac systems, to the extent that Bcl11b knockout mice are incompatible with extra-uterine life. Bcl11b has been implicated in a number of disease states including Huntington's disease, Alzheimer's disease, HIV and T-Cell malignancy, amongst others. Bcl11b is a fascinating protein whose critical roles in the CNS and other parts of the body are yet to be fully explicated. This review summarizes the current literature on Bcl11b and its functions in development, health, and disease as well as future directions for research.

## Bcl11b: structure and function

B cell leukemia 11b (BCL11b), also named Radiation induced tumor suppressor gene 1 (RIT1) and Coup-TF interacting protein 2 (CTIP2) and is a Kruppel-like C_2_H_2_ zinc finger protein transcription factor. The encoding gene, present in human chromosome 14, was initially found to be a tumor suppressor gene closely related to Bcl11a, a proto-oncogene (Satterwhite et al., 2001). Bcl11b has alpha and beta splice isoforms which comprise 823 (89.1 kDa) and 894 (95.5 kDa) amino acids respectively in humans. The beta isoform includes Exon 3 whereas this is spliced out in the alpha but both contain Exon 4 which includes the six critical C_2_H_2_ zinc finger binding domains and thus are functionally similar (Lennon et al., 2016). Of those, domains 3, and 4 bind to the DNA, while other regions are important for protein interactions. The murine Bcl11b gene is 88% similar to the human gene and is on chromosome 12 rather than 14 (Huang et al., 2012). In mice there are alpha, beta and gamma isoforms of Bcl11b, 75, 89.4, and 97.1 kDa, respectively, with the beta form being the most highly expressed (Desplats et al., 2008). Bcl11b acts both directly, by attaching to promoter regions, and indirectly, by binding to transcription factors bound to promoter regions themselves (Cismasiu et al., 2005). In its role as a transcription factor Bcl11b acts as both a repressor and activator of a multiplicity of genes. For example Bcl11b interacts with the orphan nuclear receptors known as chicken ovalbumin upstream promoter transcription factors COUP-TF (Avram et al., 2000) and nucleosome remodeling and histone deacetylation complex (NuRD; Cismasiu et al., 2005) to act as a powerful genetic repressor for a number of genes. As a converse example, Bcl11b and p300 co-activate at upstream site 1 in the IL-2 promoter causing transactivation of IL-2 expression in activated T lymphocytes (Cismasiu et al., 2009).

## Neuronal expression of Bcl11b in development and adulthood

Leid et al. (2004) characterized Bcl11b and Bcl11a expression throughout murine development at E10.5, 12.5, 14.5, 18.5, P21, and adult mouse brain. They found that at E10.5 Bcl11b was expressed diffusely through the embryo. Through 12.5 and 14.5 the CNS expression localized to layers IV/V of the cortex, limbic system, olfactory bulb, basal ganglia, and CA1/2 regions of the hippocampus (Leid et al., 2004). From E18.5 to P21 Bcl11b expression was maintained in the cortex, hippocampus and basal ganglia and this pattern of expression remained through adulthood. This selective distribution of Bcl11b through the CNS underlies its role in specification and differentiation, and is further discussed below.

### Role in corticospinal motor neurons

Bcl11b directs pathfinding and development of axons in Corticospinal Motor Neurons (CSMNs), which are 1st order motor neurons that run in the pyramidal tracts providing descending control from the cortex to the spinal motor neurons (Chen et al., 2004). Bcl11b is highly expressed in CSMNs of cortical level V but is absent from cortico-cortical neurons, which are characterized by Satb2 expression prenatally (Fame et al., 2012; Harb et al., 2016). Chen et al. discovered that Fezf2 works upstream of Bcl11b and determines whether neocortical neurons project to the cortex or subcortically (Chen et al., 2008). Bcl11b-knockout mice die on day 1 after birth demonstrating irregularities in uniform bundling, growth and pathfinding of CSMN axons. Heterozygote Bcl11b^+/−^ mice manifest decreased ability to functionally prune and regulate axons, albeit to a lesser extent. It was thought to be a specific marker of layer V and VI neurons but has recently been found to be highly expressed in GABAergic interneurons in layers I—VI (Arlotta et al., 2005; Nikouei et al., 2016). Embryologically the difficulty for CSMNs is to direct axonal projections over significant distances to specific locations in the spinal gray matter. Arlotta et al. (2005) found that on immunohistochemistry of wild type mice at E18, P3, P6, and P14 Bcl11b was co-expressed with Netrin-G1, csmn1, Cadherin 13, and Cadherin 22, which are known to functionally direct axonal projections and delineate CSMNs from callosal projection neurons (Abbas et al., 2014). The Notch pathway controls the transcription of these pathfinding proteins. Bcl11b was identified as an interacting partner and regulator of the Notch signaling pathway in T cell lines (Yatim et al., 2012) but as yet this has not been confirmed in neurons.

### Role in cortical gabaergic interneurons

Bcl11b is present in the majority of cortical GABAergic interneurons. Nikouei et al. (2016) found that almost all Bcl11b positive staining cells in the somatosensory cortex layers I-IV of adult mice were GABAergic interneurons. Interestingly in layer V, where Bcl11b was thought to be specific for subcerebral projection neurons it was found that almost 40% of Bcl11b staining cells were interneurons. Indeed on single cell mRNA sequencing the interneurons demonstrated significantly higher expression of Bcl11b compared to subcerebral projection neurons which complicates it's previous use as a marker for CSMNs. Amongst the subclasses of GABAergic interneurons Bcl11b was expressed in 64% of parvalbumin expressing interneurons, 73% of somatostatin expressing neurons and 42% of 5HT3 ligand gated ion channel receptor-expressing neurons indicating that it may have a role in subtype specification of interneurons. It's may also play a role in directing local axonal projection but its function in these cells has not been fully explored (Nikouei et al., 2016).

### Role in striatal medium spiny neurons (MSNs)

Bcl11b has an integral role in the development and maintenance of striatal inhibitory GABAergic MSNs which comprise 90–95% of the striatum (Gerfen, 1992; Graybiel, 2005). Arlotta et al found in adult wild type mice within the striatum it consistently co-localized with DARPP-32 and was consistently excluded from somatostatin and ChAT expressing cells indicating its specificity for MSNs and absence from striatal interneurons. They also found striatal expression was highest anteriorly and lowest posteriorly suggesting that it has a role in patterning and structuring the striatum (Arlotta et al., 2008). Bcl11b is expressed in the immature migratory cells in the mantle zone of the striatum from E13.5 but is excluded from the dividing progenitors of the SVZ/VZ in the lateral ganglionic eminence where MSNs are generated. Bcl11b expression increases as the MSNs migrate and mature in the striatum and is maintained throughout adulthood. All these data corroborate the idea that Bcl11b is critical to the terminal differentiation and maintenance of MSNs rather than early neurogenesis.

Indeed in Bcl11b^−/−^ knockout mice MSNs fail to differentiate, form normal cellular patch-matrix collections and repel heterotopic cellular aggregates from invading into the striatum, resulting in abnormal dopaminergic innervation and a dysfunctional striatum (Arlotta et al., 2008). Interestingly, researchers have produced MSNs from adult fibroblasts by the co-expression of miRNA 9/9^*^, 124 and transcription factors Bcl11b, Dlx1, Dlx2, and Myt1l. When implanted into mouse striatum the human cells persisted *in situ* for greater than 6 months and extended projections to normal targets, opening exciting possibilities for its future utilization for neuroregenerative stem cell therapies (Victor et al., 2014).

ChIP-Seq transcriptome experiments of cultured striatal cell lines indicate that Bcl11b is a regulator of a myriad of genes of the Brain Derived Neurotrophic Factor (BDNF) signaling pathway (Tang et al., 2011). Tang et al. (2011) found that there were seven genes targeted by Bcl11b within the pathway including: Rps6ka5, Irak4, Plcg2, Mapk9, Shc1, Mapk10, and Rapgef1 as well as targeting BDNF. Interestingly there were also six target genes in the Epidermal Growth Factor (EGF) pathway including: Nrg3, Pak4, Plcg2, Mapk9, Shc1, and Mapk10. The gross preponderance of changes in expression for both pathways were decreases, indicating that Bcl11b is a negative regulator of BDNF and EGF signaling. The BDNF signaling pathway is critical to neuroplasticity and development of higher-order cortical functions such as learning and memory. Thus, dysregulation of neurotrophins, particularly BDNF, have been implicated in a myriad of neurodegenerative diseases, including Alzheimer's, Huntington's and Parkinson's diseases (Zuccato and Cattaneo, 2009). Specific targeting of Bcl11b expression and function could represent a novel therapeutic approach to modifying BDNF signaling specifically in striatal cells.

### Role in hippocampus

Bcl11b is highly expressed in the hippocampus, serving an axial functions in both development of and adult neurogenesis in the dentate gyrus (Simon et al., 2012). During development Bcl11b is present in the hippocampus of mice at E15 starting in the CA and extending into the suprapyramidal blade of the dentate gyrus at E18. Postnatally and extending into adulthood it is present in the CA1/CA2 regions as well as post-mitotic granule cells of the dentate gyrus. Forebrain specific Emx-Cre Bcl11b^flox/flox^ mice show 33% less cells, poorer cellular structure, smaller cells and immature cellularity in the dentate gyrus demonstrating its roles in early neurogenesis, differentiation and structural organization (Simon et al., 2012).

The dentate gyrus of the hippocampus is one of only two neurogenic stem cell niches that persist into adulthood—the other being the lateral ventricles of the subventricular zone. In adult mice injected with BrdU, a marker of cell proliferation, and then stained with markers of mature granule cells (NeuN and Calbindin) the adult-born neurons remain in undifferentiated, early post mitotic stages, confirming a role of Bcl11b in proliferation and neuronal maturation. Additionally, Simon et al found that induced Bcl11b ablation in adult mice caused a reduction in dentate gyrus size and cellularity, and increased apoptosis after as little as 2 months. Taken together, these data indicate Bcl11b is critical for adult neurogenesis specifically the specification, maintenance, and neuronal integration of new adult born hippocampal granule cells as well as being necessary for the survival of mature granule cells (Simon et al., 2016). Consequently Bcl11b^flox/flox^ mice have impaired learning, memory, and maze solving skills. Interestingly Simon et al found that a similar phenotype was present in desmoplakin forebrain-specific mutant mice and re-introduction of desmoplakin cDNA to the Bcl11b forebrain mutants returned neurogenesis to normal. They posit that desmoplakin, an intercellular adhesion molecule, is a critical effector of Bcl11b in the hippocampus (Simon et al., 2012).

Given that the dentate gyrus is one of the two regions of adult neurogenesis in the brain and that the hippocampus is critical to spatial learning and memory formation, loss of Bcl11b may have critical roles in neurodegenerative disease. Bcl11b has been observed as a target of the mir-17 cluster (Lewis et al., 2003; Zeller et al., 2003) as well as miR-93 and let-7 in T cells (Kurosawa et al., 2013). Given the accumulating data of the importance of Bcl11b in critical brain regions such as hippocampus, the expression of microRNAs targeting Bcl11b in the hippocampus is a fertile area for further study.

### Role in vomeronasal sensory neurons

The Vomeronasal organ is present and functioning in mice in humans it is present during fetal development but regresses before birth and is present as a non-functioning remnant in a portion of adults (Kjaer and Fischer Hansen, 1996; Meredith, 2001; Trotier, 2011). Within mice the Vomeronasal system Bcl11b is found in post mitotic vomeronasal sensory neurons (VSNs) of the vomeronasal epithelium (VNE) in addition to projection neurons and GABAergic interneurons in the accessory olfactory bulb. Enomoto et al demonstrated that in wild type mice Bcl11b is first observed within the vomeronasal groove at E11.5. The expression increased through E16.5 to P0 but after birth gradually diminished and was limited to the marginal region of the VNE, an area of precursor cells and immature neurons. On immunohistochemistry they found that Bcl11b at P0 and P14 co-localized with GAP43, SCG10 and NeuroD which are markers for immature neurons and differentiating/post mitotic neurons. It was excluded from Mash1 and OMP staining cells which are markers for vomeronasal neuronal precursors and mature neurons respectively indicating that Bcl11b has a role in the early differentiation/immature neurons. Interestingly in Bcl11b^−/−^ mice cells are produced in sufficient numbers but subsequently VSNs undergo apoptosis selectively. Similar to irregularities in the mice Bcl11b deficient motor cortex, the vomeronasal system manifests disoriented laminar structure, impaired axonal projections of VSNs, down-regulation of vomeronasal receptor genes, and thus undifferentiated VSNs (Enomoto et al., 2011). Thus Bcl11b is critical in the differentiation, specification, and structural organization in VSNs.

## Other roles

Bcl11b is critical for the specification and differentiation of αβ T cells. Bcl11b knockout precursor T cells demonstrate an arrest of differentiation at an immature DN2 stage (CD4-, CD8-; Liu et al., 2010; Kominami, 2012) but no changes in the development of cells of B- or γδ T cell lineages. In these cell lines the lack of differentiation is recognized by the p53 pathway and leads to profound apoptosis of the Bcl11b^−/−^ T cells (Okazuka et al., 2005; Karanam et al., 2010; Zhang et al., 2010). Conversely naïve T cells overexpressing Bcl11b manifest increased proliferation, most notably in the Th group (Ikawa et al., 2010; VanValkenburgh et al., 2011). Recently it has been found that Bcl11b induces mammary stem cells into the G0 cell cycle phase, entering quiescence (Cai et al., 2017). Similar to maintenance of mammary epithelium Bcl11b has an important role in epidermal stem cell maintenance and integrity as well as sphingolipid biosynthesis (Ganguli-Indra et al., 2009; Wang et al., 2012a; Bollag, 2013; Inoue et al., 2016). Bcl11b functionally directs mammalian odontogenesis and is crucial for the morphogenesis of lingual papilla and differentiation of oral epithelium (Kyrylkova et al., 2012; Nishiguchi et al., 2016).

## Involvement in pathology

### Huntington's disease

Huntington's disease (HD) is marked by the ubiquitous pathological expression of the Huntingtin protein with toxic neurodegenerative changes predominant in the striatum (Ross and Tabrizi, 2011). Bc11b is significantly diminished in HD cell lines, mouse models and human CSF and tissue samples. Ahmed et al suggest that the toxic effects of Bcl11b deficiency are mediated by decreases in inositol polyphosphate multikinase (IPMK; Ahmed et al., 2015). IPMK, a kinase and a transcriptional co-activator, is severely diminished in Bcl11b knockout cells lines and is returned to normal levels when Bcl11b is replaced. As with Bcl11b IPMK is reduced in HD cell lines and mouse models and indeed it was found in R6/2 HD mouse model that virally transfected intrastriatal IPMK delivery prevents the progression of psychomotor dysfunction and reduces striatal pathology. Similarly Desplats et al found that the deleterious impacts of Huntingtin protein in the Q111 cell line are diminished when Bcl11b is expressed at supranormal levels (Ahmed et al., 2015). Reversing the loss of Bcl11b-IPMK expression is therefore a promising novel therapeutic target in HD. Bcl11b attaches to the proximal promoter region a multiplicity of striatally expressed genes including *Pde10a, Isl1 Klf9, and Dgke*. Desplats et al posits that the deficiencies of the normally striatally enriched genes are in part caused by Bcl11b insufficiency and underlie the neuropathology of HD (Desplats et al., 2008). Given it's role in a number of neurodegenerative diseases putative linkages to motor neuron disease has also recently been discussed (Lennon et al., 2016).

### Alzheimer's disease

Like HD, Alzheimer's is a neurodegenerative disease marked by the pathological presence of B amyloid plaques and tau protein found in neurofibrillary tangles (Braak and Braak, 1991). A recent genome wide association study of 41 families with hereditary late onset Alzheimer's disease (LOAD) identified Bcl11b as a novel gene defect involved. They suggest a pathological pathway involving Bcl11b's negative regulation of brain-derived neurotrophic factor (BDNF) signaling, a crucial pro-survival and neuroprotective cytokine. Aberrant BDNF signaling is involved in several neurodegenerative diseases, including LOAD, in which low levels have been associated with worsening disease and higher levels have been shown to be protective (Nagahara et al., 2009; Weinstein et al., 2014). Thus BDNF-based therapies have been proposed for Alzheimer's and other neurodegenerative disease. Kunkle et al suggests that targeting of Bcl11b-BDNF interactions may be a potential therapeutic approach to increasing levels of BDNF (Kunkle et al., 2016).

### NeuroHIV

Bcl11b has been strongly associated with latent neuroglial reservoirs of HIV-1. Desplats et al demonstrated that Bcl11b was raised in latent HIV patients CSF. There increases were closely correlated with elevations of HDAC1, MeCP2, and HP1-alpha. Each of these act as transcriptional HIV silencers, through processes of histone modification. Post-mortem tissue immunostaining analysis found Bcl11b present in both astrocytes and microglia and CSF immunoblot found that it was significantly elevated compared with both healthy and non-latent HIV controls (Desplats et al., 2013). However, other potential reasons for Bcl11b elevation such as inflammation and neurodegeneration were not explored. Bcl11b has been found to have two actions in the suppression of latent HIV-1 (Lennon et al., 2016). In cultured human microglial cell lines Bcl11b, acts with the NuRD co-repressor complex to downregulate HIV-1 LTR transcription, thus diminishing HIV TAT expression, a critical regulator of viral transcription (Marban et al., 2005). Furthermore, Bcl11b downregulates HIV-1 reactivation when TAT is not present and thus it also inhibits endogenous transcription factors including P-TEFb (Cherrier et al., 2013). Bcl11b and NuRD co-repressor complex causes heterochromatin development and deacetylation of histones in the LTR HIV1 sequence and thus transcriptionally silences it (Cismasiu et al., 2008). This promoter region heterochromatin formation induces HIV-1 latency and may similarly induce latency in endogenous retroviruses (Le Douce et al., 2014a; Lennon et al., 2016).

### Other pathologies

Bcl11a and 11b were first discovered by Satterwhite et al., 2001 when they found a translocation of t(2:14) (p13;q32.1) B cell malignancies causing dysfunction of Bcl11a, an immunoglobulin heavy chain gene, and its close relative Bcl11b (Satterwhite et al., 2001) with deletions or missense mutations present in 9% of mature T-ALL specimens (Kamimura et al., 2007; Gutierrez et al., 2011). Bcl11b is a suppressor of apoptosis with knockout cells showing apoptotic pathway activation, mitochondrial membrane potential loss and elevation of BclxL, Caspase 8, and caspase 9. (Karanam et al., 2010). Bcl11b is integral for T cell differentiation, in particular T cell identity and VDJ recombination (Liu et al., 2010; Li et al., 2013). Bcl11b complexes with Notch1 proteins to facilitate V(D)J recombination and express TCR-β, a key step in αβ T cell differentiation (Rothenberg and Scripture-Adams, 2008). Ultimately Bcl11b-deficient T cells are more undifferentiated and thus have greater proliferative and oncogenic potential (Nagel et al., 2007; Zhang et al., 2012). Fascinatingly a hypofunctioning, missense mutation of Bcl11b has recently been found to be the causative genetic defect in a child with severe combined immunodeficiency, in which the child also demonstrated developmental arrest of immature T cells, absence of a corpus callosum and dermal, craniofacial abnormalities, mental retardation and impaired acquisition of language (Punwani et al., 2016). Interestingly Bcl11b has also very recently been connected to neuropsychiatric disease by Whitton et al. who identified Bcl11b as one of seven genes in which polymorphisms were associated with schizophrenia. However, there was no correlation of Bcl11b with any of the cognitive functional outcomes tested (IQ, working memory, episodic memory, and attention) and thus the connection between Bcl11b and schizophrenia presently remains unclear (Whitton et al., 2016).

VanValkenburgh et al. demonstrated by knockdown of Bcl11b at the T cell double-positive stage caused inflammatory bowel disease in mice because of a dysregulation of proinflammatory cytokine production in CD4+ T cells infiltrating the colon (VanValkenburgh et al., 2011). Wang et al. found a similar occurrence in the skin, in which a dermatitis-like skin inflammatory response was elicited when there was selective ablation of the Bcl11b in keratinocytes (Wang et al., 2012b). Bcl11b deficiency has also been associated with dysregulation of intracellular signaling in cardiomyocytes resulting in hyperproliferation and hypertrophic cardiomyopathy (Le Douce et al., 2014b).

## Conclusion

Bcl11b is a complex multifunctional protein with important roles neuronally and non-neuronally. When using Bcl11b as a marker for neurons it is important to keep in mind it's alternative sources as well as that it is expressed in a multiplicity of different subtypes of neurons even within the same cerebral cortex layers. It has essential roles in development and its deficiency and dysregulation results in death and various disease states.

## Author contributions

MJL: Performed majority of drafting work and literature search. SJ: Provided direction to content and research. Provided significant editorial support. MDL: Provided direction to content and research. Provided significant editorial support. GG: Provided direction to content and research. Provided significant editorial support. BB: Head of lab, provided impetus, and structural framework for research. Provided significant editorial support.

## Funding

The authors acknowledge the generous support and funding of the Peter Duncan Neurosciences Research Unit and a University of New South Wales (UNSW) Goldstar grant for this study. Neither of these agencies influenced the decision to publish.

### Conflict of interest statement

The authors declare that the research was conducted in the absence of any commercial or financial relationships that could be construed as a potential conflict of interest.
